# Bending It Like Beckham: How to Visually Fool the Goalkeeper

**DOI:** 10.1371/journal.pone.0013161

**Published:** 2010-10-06

**Authors:** Joost C. Dessing, Cathy M. Craig

**Affiliations:** 1 Center for Vision Research, York University, Toronto, Ontario, Canada; 2 Research Institute MOVE, Faculty of Human Movement Sciences, VU University Amsterdam, The Netherlands; 3 Canadian Action and Perception Network (CAPnet), York University, Toronto, Ontario, Canada; 4 School of Psychology, Queen's University Belfast, Belfast, Northern Ireland, United Kingdom; CNRS, France

## Abstract

**Background:**

As bending free-kicks becomes the norm in modern day soccer, implications for goalkeepers have largely been ignored. Although it has been reported that poor sensitivity to visual acceleration makes it harder for expert goalkeepers to perceptually judge where the curved free-kicks will cross the goal line, it is unknown how this affects the goalkeeper's actual movements.

**Methodology/Principal Findings:**

Here, an in-depth analysis of goalkeepers' hand movements in immersive, interactive virtual reality shows that they do not fully account for spin-induced lateral ball acceleration. Hand movements were found to be biased in the direction of initial ball heading, and for curved free-kicks this resulted in biases in a direction opposite to those necessary to save the free-kick. These movement errors result in less time to cover a now greater distance to stop the ball entering the goal. These and other details of the interceptive behaviour are explained using a simple mathematical model which shows how the goalkeeper controls his movements online with respect to the ball's current heading direction. Furthermore our results and model suggest how visual landmarks, such as the goalposts in this instance, may constrain the extent of the movement biases.

**Conclusions:**

While it has previously been shown that humans can internalize the effects of gravitational acceleration, these results show that it is much more difficult for goalkeepers to account for spin-induced visual acceleration, which varies from situation to situation. The limited sensitivity of the human visual system for detecting acceleration, suggests that curved free-kicks are an important goal-scoring opportunity in the game of soccer.

## Introduction

In the game of soccer, the free-kicks scenario has become an increasingly important opportunity to score a goal. Although helped by innovations in shoe and soccer ball design, it can also be attributed to the free-kick specialists (e.g., David Beckham, Juninho, and Keisuke Honda), who skilfully apply spin to the ball at shoe-ball contact causing the resulting ball trajectory to bend. Why do players do this? Do spin-induced deviations from a standard parabolic trajectory make it more difficult for a goalkeeper to intercept the ball? Is the spin fooling the goalkeeper, much like curved pitches fool baseball batters [1]? While the perceptual effects of ball spin have been documented before [2], [3], here we directly examine and explain the type of movements goalkeepers make as they attempt to stop curved and standard parabolic free-kicks.

**Figure 1:**
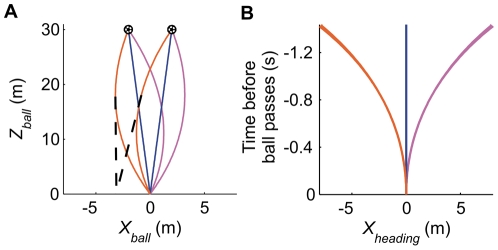
Heading direction of straight and curved free kicks. (A) Top view of a subset of the used ball paths (orange = counter-clockwise spin [CCS]; blue = no spin [NS]; pink = clockwise spin [CS]). The dotted lines represent the ball's heading direction at 0.5 s. If the ball were to continue with this heading direction for these exemplary trajectories, the ball would pass the goal line at −3.1 m. The ball's instantaneous heading location (*X_heading_*) is equal to 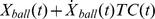 (with 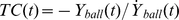), which does not take lateral ball acceleration into account. (B) *X_heading_* as a function of time before the ball passes the goal line. If goalkeeping movements are aimed towards the ball's heading location, and thus do not take lateral ball acceleration into account, movements would be biased in a leftward direction for CCS free-kicks (involving rightward acceleration) and in a rightward direction for CS free-kicks (involving leftward acceleration).

A ball that spins around an axis other than its motion axis experiences a force perpendicular to the direction of travel, deflecting it from its standard parabolic trajectory [2]–[4]. For free-kicks in soccer, this deflection predominantly amounts to a lateral acceleration. Curved free-kicks may be especially difficult to handle, even for professional goalkeepers, because the human visual system is not very sensitive to acceleration [2]–[12]. Although humans may have internalized the effects of gravitational acceleration, which remains constant across conditions [13]–[15], this is not viable for spin-induced acceleration that varies from situation to situation. If goalkeepers *do not* fully take the lateral ball acceleration into account when controlling their movements online, we predict that their movements should be biased in the direction of initial ball heading, away from the spin-induced lateral ball acceleration (Figure 1) [16], [17]. Conversely, if they *do* take it into account their movements should be directly aimed at ball arrival position. We tested these predictions by analyzing hand movements in a goalkeeping experiment.

**Figure 2:**
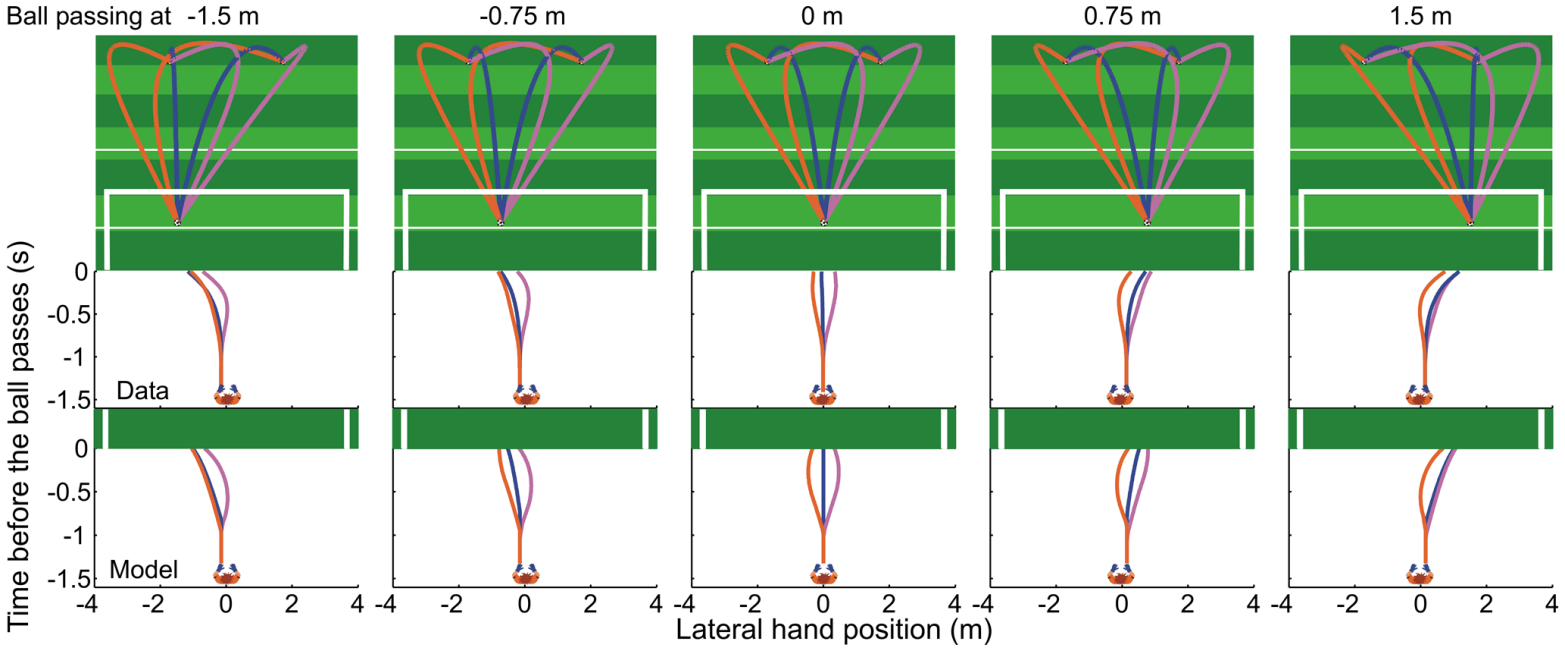
Three-dimensional ball paths and corresponding observed hand movements. Spin direction (curve) is represented using different colours (orange = counter-clockwise spin [CCS]; blue = no spin [NS]; pink = clockwise spin [CS]). The middle panels depict the lateral hand position as a function of the time before the ball passes the goal line, averaged across 10 trials (five repetitions for two initial ball positions) and 10 novice participants. The bottom panels depict the simulated movements for the same conditions as predicted by our goalkeeper model, averaged across the two initial ball positions.

Studying goalkeeper behaviour in the real world is nigh-impossible due to the difficulty in reproducing the same ball trajectory across trials. Virtual reality (VR) can provide a viable alternative, particularly if the user's interactions with the virtual world mirror those found in the real world. Significant improvements in processing speeds, graphics and motion tracking interfaces make this technology ready to deliver on its promises to behavioural neuroscience [18]. We created a goalkeeping task using immersive, interactive VR that afforded natural body and hand movements. Twelve participants (including two expert, professional goalkeepers) attempted to stop free-kicks by controlling the position and orientation of virtual depictions of their hands (Movie S1 and Movie S2). Free-kicks with different spin directions (clockwise [CS], counter-clockwise [CCS] and no spin [NS]) were used, passing the goal line at different lateral positions (Figure 2). Initial ball position was also varied, but not analyzed statistically.

## Results

### Novice goalkeepers

**Figure 3:**
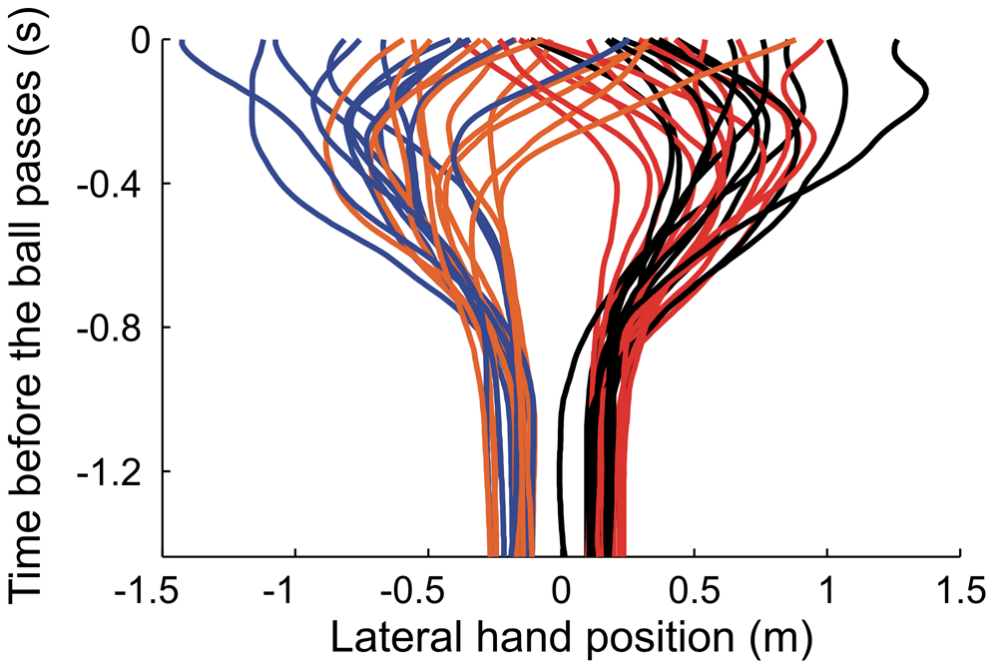
Initial erroneous movements in goalkeeping. For each participant (including experts) this figure shows the lateral hand position as a function of the time before the ball passes the goal line for the single most extreme initial erroneous movement for four ball trajectories (red = clockwise spin passing at −1.5 m; black = clockwise spin passing at −0.75 m; orange = counter-clockwise spin passing at 1.5 m; blue = counter-clockwise spin passing at 0.75 m).

Figure 2 illustrates how hand movements depend on the ball's spin direction (i.e., curve) and passing position. Curved and straight free-kicks were stopped with similar movements if they passed on the side to which they initially appeared to move, while movements appeared to be affected by spin-direction if the ball initially headed to the side opposite to which it would actually pass (e.g. CCS to 0.75 m or 1.5 m; CS to −0.75 m or −1.5 m). For these free-kicks the hands were often initially moved in the wrong direction, up to 1.0 m in some trials (Figure 3). This initial erroneous movement leaves more distance to cover to get to where the ball will eventually pass, while the remaining time to cover that extra distance is even shorter. This should translate into greater final errors, which would affect performance. Indeed, our novice participants only stopped 6% of these free-kicks, as opposed to 56% of the other free-kicks.

These effects were quantified using the early bias and final error in the hand movements. These were defined as the amplitude of initial erroneous movements (early bias) and the difference between the lateral hand and ball position as the virtual ball passed the hand in depth (final error). The analysis of recorded hand movements showed that both were substantially biased in the direction of initial ball heading (i.e. the opposite direction of the spin-induced lateral ball acceleration). This meant that initial hand movements were leftward for CCS free-kicks and rightward for CS free-kicks (early bias: *F*_2,18_ = 54.08; *P*<10^−7^; final error: *F*_2,18_ = 38.28; *P*<10^−6^; all post-hoc comparisons *P*<0.0005). Goalkeeper movements therefore appear to be influenced by the spin-induced force that deflects the ball from a standard parabolic trajectory, confirming the hypothesis that goalkeepers do not fully account for lateral ball acceleration when controlling their movements online.

**Figure 4:**
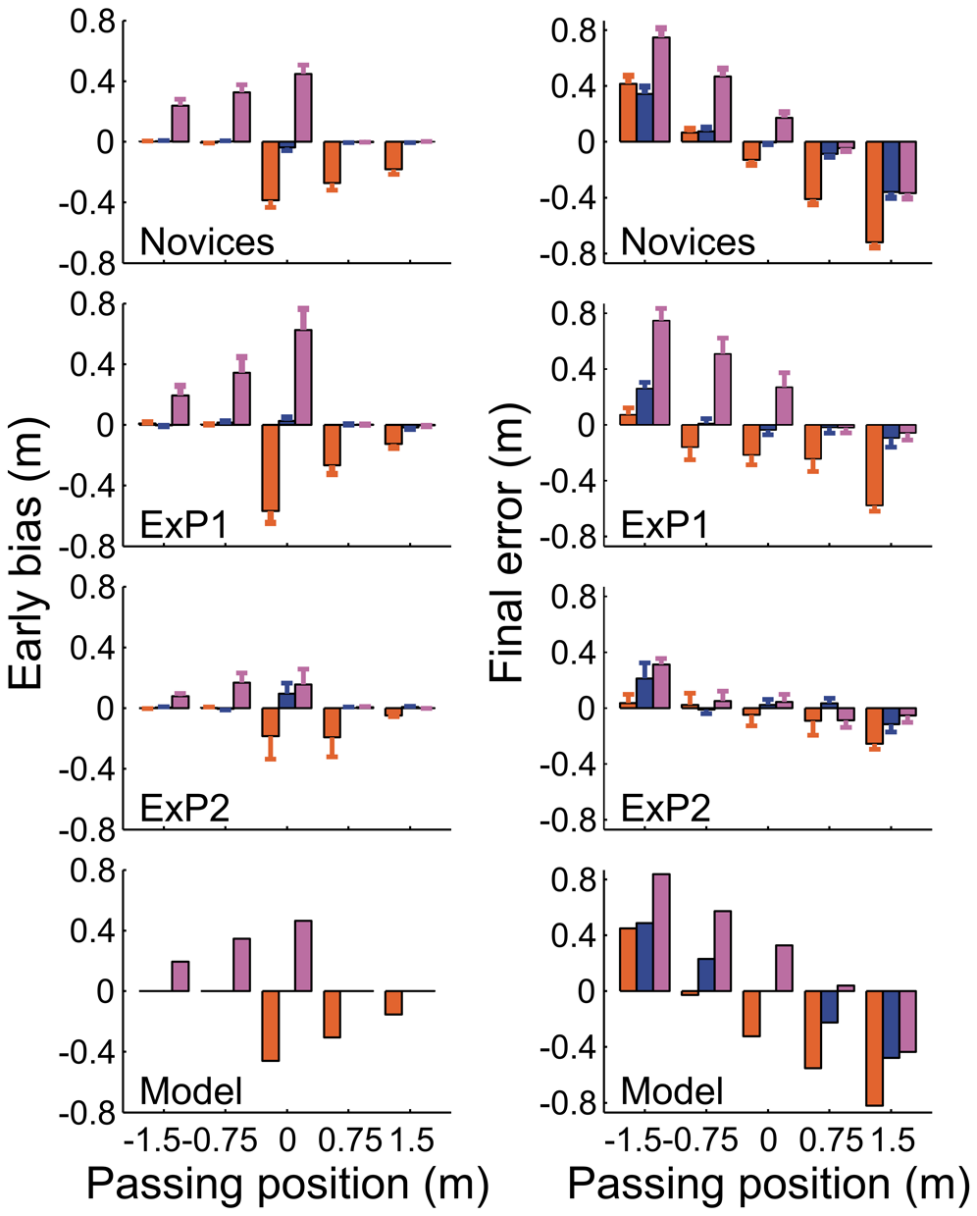
Movement biases in goalkeeping. Left and right panels show the early bias and final error, respectively, broken down as a function of the passing position and curve direction (orange = counter-clockwise spin [CCS]; blue = no spin [NS]; pink = clockwise spin [CS]). Top panels show the averaged data for the novices; values are averaged over 10 trials (five repetitions for two initial ball positions) and 10 participants. Error bars show standard errors across participants. The second and third rows depict the averages for ExP1 and ExP2, respectively, where error bars indicate standard errors across the 10 trials. The bottom row indicates the values calculated from the model simulations (see Figure 2).

While the movements were also influenced by passing positions (early bias: *F*_4,36_ = 28.60; *P*<10^−10^; all *P*<0.05; final error: *F*_4,36_ = 176.72, *P*<10^−15^; all *P*<0.0001), these effects are particularly telling in the interaction with spin direction (early bias: *F*_8,72_ = 48.40; *P*<10^−15^; final error: *F*_8,72_ = 25.21; *P*<10^−15^; Figure 4), which shows that the direction of spin did not influence the movements symmetrically for all passing positions. For those curved free-kicks that elicited an initial erroneous movement, the early bias increased in magnitude towards the goal centre; for NS free-kicks the early bias was very small and uninfluenced by passing position. The early bias did not differ significantly between CS free kicks passing at −0.75 m and 0 m and between those passing at 0.75 m and 1.5 m; for CCS free kicks the early bias was similar for balls passing at −1.5 m and −0.75 m and for those passing at 0.75 m and 1.5 m (all other post-hoc comparisons *P*<0.001). The final error did not differ significantly between some curved and straight (NS) free-kicks; this concerned CCS free-kicks passing at −1.5 m and −0.75 m and CS free-kicks passing at 0.75 m and 1.5 m (Figure 2). The final error also did not differ significantly between NS ball trajectories passing at 0 m and −0.75 m; all other post-hoc comparisons *P*<0.01; Figure 4). This interaction highlights asymmetric effects of spin for curved ball trajectories which were initially heading to the side of the goal where they would not actually pass. It further suggests that the observed movement biases were influenced by factors additional to an inability to account for lateral ball acceleration, an issue that will be addressed in more detail in the goalkeeper model presented later.

Goalkeepers may adapt their movements to minimize the effects of spin. For instance if they waited longer before initiating a movement, thus observing the ball trajectory longer [19], they might improve their ability to detect how the ball is curving (e.g., extracting visual ball acceleration from velocity changes over time [20], [21]). This could lead to reduced movement biases for curved free-kicks. To check if such a strategy might have been used, we assessed whether movement initiation time (relative to the start of ball motion) also varied with spin direction and passing distance. Here, we discuss the findings for the novices.

**Figure 5:**
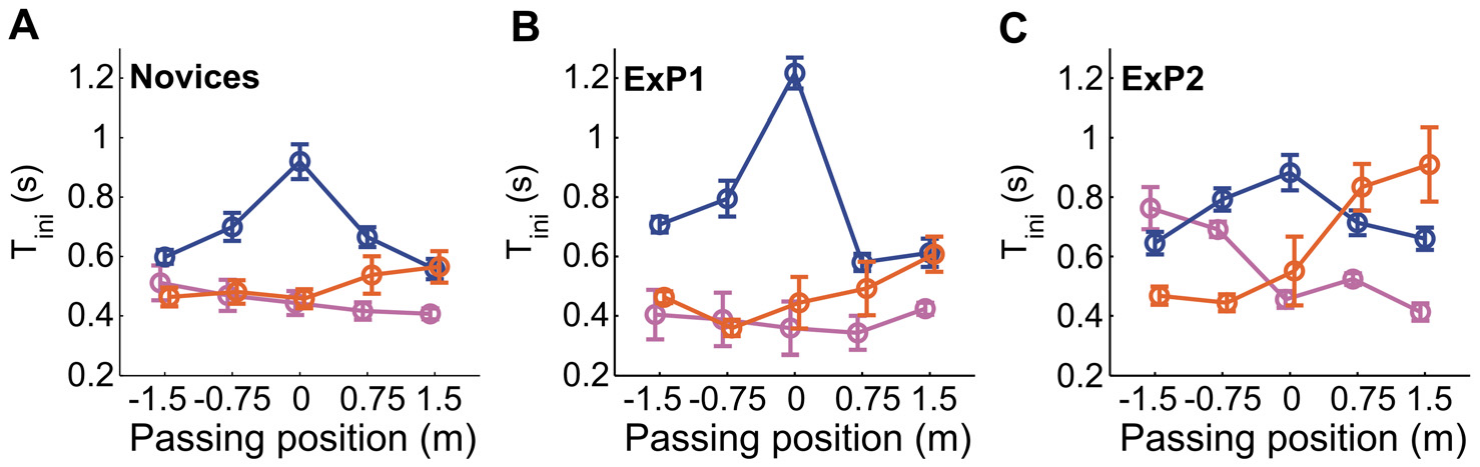
Initiation times for all participants. (A) Moment of initiation (T_ini_) for the novices as a function of the passing position and curve (orange = counter-clockwise spin [CCS]; blue = no spin [NS]; pink = clockwise spin [CS]). Values were averaged across 10 trials (5 repetitions and two initial ball positions). Error bars show standard errors across participants. (B) Same as (A), but now for ExP1, where error bars indicate standard errors across the 10 trials. (C) Same as (B), but now for ExP2.

The novices were found to initiate their movements significantly later for NS free-kicks than for both CCS free-kicks and CS free kicks (*F*_4,36_ = 41.00; *P*<10^−6^; all post-hoc comparisons *P*<0.05) and for balls passing at 0 m compared to those passing at −1.5 m and 1.5 m (*F*_4,36_ = 6.98; *P*<0.001; both post-hoc comparisons *P*<0.005). These effects were also found to interact (*F*_4,36_ = 10.76; *P*<10^−9^; Figure 5). Movement initiation occurred significantly later for NS free-kicks arriving at the centre of the goal (0 m), compared to those passing at all other positions (all *P*<0.005). Moreover, goalkeeper movements were initiated later for straight (NS) compared to curved free-kicks passing at 0 m, and earlier for CS than for NS free-kicks, when they passed at 0 m, 0.75 m and 1.5 m (all *P*<0.001). These effects illustrate that while novices scaled their movement times for the distance to be covered for the NS free-kicks trials, they did not do so for curved free kicks.

### Expert goalkeepers

Previous studies have shown that expert goalkeepers have similar perceptual difficulties as novices in judging where curved free-kicks will go [2], [3]. While the visual system may constrain expert performance, it is not a given that actual movements of expert goalkeepers reflect their perceptual difficulties. As mentioned above, variations in movement initiation time may afford indirectly accounting for lateral ball acceleration. Particularly the superior motor control of experts may allow them to observe the ball trajectory longer and better detect the curve of the ball trajectories before initiating their better aimed movements. This would reduce their movement biases for curved free-kicks.

ExP1 did not confirm this prediction, showing movement biases and movement initiation times that were very similar in direction and magnitude to that found for the novices (Figures 4 and 5). This may be related to the fact that this expert has yet to experience competitive soccer at an adult national league level, having played for his country only at junior level (Under 16s). ExP2 - the more experienced goalkeeper - on the other hand indeed showed substantially smaller movement biases (Figures 4 and 5). This suggests that ExP2 accounted better for the spin-induced curve than the novices did. Most likely, his additional years of experience playing top-level soccer, meant that he had learned to extract acceleration signals from velocity changes over time [20], [21]. Alternatively, he may have become better at predicting the curved shape of the trajectory [22], which would not specifically require the use of acceleration signals. In particular, ExP2 was found to wait significantly longer before initiating movement for CS trajectories passing on the left and CCS trajectories passing on the right, exactly those conditions for which the novices and ExP1 showed large initial movement biases. Thus, the later initiation may have contributed to the reduced initial movement biases ExP2 displayed in these conditions (as explained above). Nevertheless, while ExP2's movement biases were smaller than those of the novices, they varied with respect to spin direction and passing distance in a similar way. This confirms that even our most experienced participant could not fully account for spin-induced curve in controlling his movements online. The statistical analyses of the movement biases of both experts are presented in Text S1.

### Goalkeeper model

To account for the observed effects of spin as well as its dependence on where the ball entered the goal, we formulated a simple goalkeeper model capturing how movements depend on properties of the free-kick. This model describes movement planning as a continuous process on the basis of visual information pertaining to the free-kick trajectory, in a manner that generalizes to all forms of interception [16], [23], [24]. In this experiment, we aimed to establish whether lateral ball acceleration is needed to account for the observed movements. Our results suggest that it is not. As a consequence, our model assumes that goalkeeper movements are continuously aimed at the ball's current heading position (*X_heading_*; Figure 1), which corresponds to a first-order estimate (i.e., not taking lateral ball acceleration into account) of where the ball will pass the goal line. Because visual information cannot be used instantaneously, we included a visual delay (*δ_vis_*) in the definition of *X_heading_*. Effectively, *X_heading_* is defined as the delayed target position (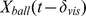) plus the (delayed) expected future displacement, given lateral ball velocity (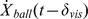) and the remaining time to ‘contact’ (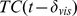):
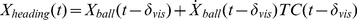
(1)

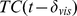
 was defined as
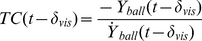
(2)Here, 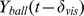 is the ball position in depth relative to the goal line and 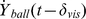 is the ball velocity in depth. It is important to note that temporal information (i.e., 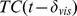) is not explicitly needed to determine *X_heading_*; substituting Equation 2 for 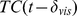 in Equation 1 (given that the time-derivative of *X* is 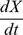) shows that *X_heading_* can also be calculated from the ball's instantaneous distance to the goal and motion direction:
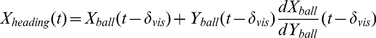
(3)
*X_heading_* can thus be calculated in several ways. Indeed, we did not aim to address the specific optical information sources that may underlie the observed goalkeeper behaviour. Our conclusions with respect to the need for information concerning lateral ball acceleration, however, do constrain the range of optical variables that may underlie the observed goalkeeper behaviour.

To model how the goalkeeper movement evolved over time, we assumed it was continuously aimed at the changing *X_heading_*; we modelled this process as a time-constrained position-servo [16], [24]:
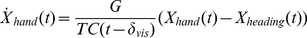
(4)Here, *G* is a gain parameter. The predicted movement was subject to a motor delay (*δ_mot_*):
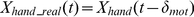
(5)


As discussed above, we observed an asymmetric effect of spin direction (Figure 4). This effect was unexpected and suggests that the observed movement biases are influenced by additional factors. We hypothesize that this observation reflects a strategy to limit spin-induced erroneous displacement. Such a strategy would come into play when the ball initially is heading to the side on which it will also finally pass (Figures 2 and 4). While this proposal is hypothetical at this stage (i.e., we did not design our experiment to test this hypothesis), we incorporated the conservative strategy of never aiming beyond a certain distance into our model to illustrate its effects on goalkeeper movements. This amounted to replacing *X_heading_* in Equation 4 with 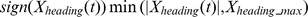:
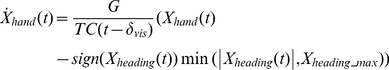
(6)Here, 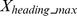 specifies the distance (on either side of goal centre) beyond which the movements will never be aimed. As can be seen from the modelled movement trajectories in Figure 2, as well as the early biases and final errors in Figure 4, our model can adequately account for the observed behavioural pattern of the novice goalkeepers, by assuming that these goalkeepers do not take lateral ball acceleration into account and that they partly compensate for this limitation by never aiming beyond a certain distance. Our model implementation illustrates how interceptive movements are continuously adjusted using visual information and how this process may be modulated by cognitive strategies.

## Discussion

We conducted a behavioural experiment in immersive, interactive VR to examine whether goalkeepers take spin-induced lateral ball acceleration into account when stopping free-kicks. Given results from previous perceptual studies [2]–[9] as well as those from neurophysiology [10]–[12], [20], we expected goalkeepers to not fully account for spin-induced lateral ball acceleration, resulting in movement biases in the direction opposite to this acceleration. Our analyses of movement biases for straight (no spin) and curved free-kicks (clockwise and counter-clockwise spin) confirmed these predictions. Even the expert goalkeepers showed erroneous initial movements, reminiscent of previous observations for ball catching [16], [17], [25]. Only the most experienced goalkeeper was able to correct for these movements, though still not completely. This confirmed the hypothesis that goalkeeper movements are influenced by the limited sensitivity to visual acceleration.

We interpreted these findings in terms of a model for movement planning in interceptive movements. In this model, goalkeeper movements at any time are aimed at the ball's current heading position, which depends only on the ball's current position and velocity (i.e., it does not account for lateral ball acceleration) [10]–[12], [20]. The observed spin-related movement biases are explained by the model much in the same way as biases during manual catching movements have been explained [16], [17], [24]. The model was based on a theory for the control of movement direction and amplitude in point-to-point reaching movements, which is consistent with neurophysiological observations [26], [27] and generalizes in a straightforward manner to different forms of interception [16], [23], [24], [28].

An unexpected finding was that the final movement error was only affected by spin direction if the ball initially appeared to head to the opposite side of the goal, compared to where it would actually pass. This was interpreted in terms of a new hypothesis, according to which goalkeepers never aim beyond a critical distance. Model simulations confirmed that such a strategy could indeed account for the asymmetric effects of spin direction across passing distances. More specifically, it reduced the movement biases for curved free-kicks that initially appeared to pass furthest from the centre of the goal (i.e., CCS free-kicks passing on the far left and CS free-kicks passing on the far right). For our experiment, the critical distance was defined as the most eccentric passing position, but in situations where the possible range of passing positions is uncertain this would correspond to the position of the goal posts. Such a strategy would prevent movements beyond the goal posts when balls initially appear to go wide. Given the hypothetical nature of this proposal, the specific origin of the asymmetrical effect of spin direction needs to be examined further in future experiments.

In light of the time available to move to intercept the free-kick, our participants were faced with a trade-off between starting to move early to reach the passing position in time to stop the ball, and waiting longer to observe a larger part of the ball flight to better anticipate where it might be heading [19]. This trade-off was evident in our analyses of movement initiation, in that the most experienced participant had apparently learned to wait longer before initiating movements, specifically for those curved ball trajectories eliciting erroneous initial movements in all the other participants. By doing so, he (ExP2) reduced the amplitude of the erroneous movements and improved his performance. Our model does not explain movement initiation (i.e., it is an input), but it does illustrate another reason (on top of the improved detection of curvature [20], [21]) why goalkeepers would benefit from waiting longer in certain situations. During the latter stages of the ball's approach its heading position better matches its actual passing position (Figure 1), which implies that movement biases should be smaller for later initiation. Delaying movement initiation therefore provides the goalkeeper with an indirect way of compensating for his limited sensitivity to visual acceleration. Nevertheless, he must continuously balance this desire to delay movement initiation with the fact that executing any movement will take time. Movement initiation should therefore not only rely on features of the ball's motion [29], but also on the to-be-performed action.

In this study, we used VR technology to examine a highly dynamic sport scenario. VR allows for a manageable and rigorous in-depth analysis that is nigh-impossible from studying observations of real-life behaviour [18]. To validate such an approach, it is imperative that our findings are generalizable to real-life sporting scenarios. To do so, we searched for real-life goal keeping examples of spin-induced errors (see Text S1) and found that indeed these examples do confirm the generalizability of the observed effects of spin direction. The asymmetry of this effect across passing distances may in principle be related to aspects of our VR set-up, such as its limited field-of-view, but similar effects have also been found for head movements with a much wider field-of-view [30]. Nevertheless, future tests of the hypothesized origin of this asymmetry could assess the effect of field-of-view in more detail.

Our findings explain why free-kick taking strategies may increase the probability of scoring a goal. Attacking teams could exploit the goalkeeper's difficulty in accounting for spin-induced deflections in the ball's trajectory by forcing him/her to rely solely on visual ball trajectory information. This can be achieved by occluding the kicker and initial ball trajectory using an additional wall of attacking players, and/or by having multiple potential kickers running up to the ball simultaneously (instead of sequentially, as is often done). The latter strategy creates uncertainty about who will actually kick the ball, thus preventing the goalkeeper from inferring the kicker's strategy before the actual kick [19].

To conclude, our results highlight how limitations of the visual system constrain movements in sports situations, even for expert athletes. Goalkeeper movements were found to be influenced by spin direction, reflecting the limited sensitivity to visual acceleration of the human visual system. Given the complexity of real-life free-kick situations, goalkeepers cannot always fully compensate for this limitation. Attacking teams would do well to exploit this whenever possible.

## Methods

### Experimental Conditions

A theoretical model incorporating aerodynamic Magnus-Robins lift and drag forces was developed (see Models). Trajectories (lasting 1.4375 s) to five different arrival positions (1.5 m high and −1.5 m, −0.75 m, 0 m, 0.75 m, and 1.5 m in lateral direction relative to the goal centre) were calculated (see Model Simulations) from two different starting positions 30 m from the goal-line and 2 m to the left and right of goal centre. For each passing position, three different spin directions were used (CS; 10 rps), counter-clockwise spin (CCS; 10 rps), and no spin (NS). The 30 resulting free-kicks (see Figure 2) were presented five times each; presentation order was fully randomised.

### Virtual Environment

A virtual soccer stadium (including lines and goal posts), conforming to FIFA regulations, was simulated using a virtual reality animation programme, 3DVia Virtools (Dassault Systems). The X, Y, Z positions and rotation of the ball, calculated using the aerodynamics model, were used to animate, in real-time, a black and white textured sphere corresponding to the colour and size of a real soccer ball (0.11 m radius) (Figure 2; Movie S2).

### Participants

Ten recreational soccer players (mean age = 29; STD = 4.7) made up the novice group. Two professional expert goalkeepers of differing levels of experience were also tested. ExP1 (age = 16) had just five years playing experience but had represented his country at international level (U-16s). ExP2 (age = 30) had over 12 years experience playing in the Irish Premier League, and has both European and International experience having played in the Intertoto, UEFA Cup and Champions League as well as representing his country. The study was approved by the School of Psychology Research Ethics Committee and adhered to the standards laid down in the Declaration of Helsinki. All participants gave written informed consent before participating.

### Apparatus

Participants viewed the virtual soccer stadium through two small screens inside a three dimensional head mounted display (HMD) unit (Cybermind Visette 45 SXGA™, 1280×1024, 60 Hz, 24 bit colour, 45° diagonal field of view,). A wireless Intersense motion IS-900 head tracker (6DOF – resolution 1.5 mm, 0.1°; recording volume 6 m×7 m×3 m – 20 Sonistrips) attached to the top of the head set was used to update in real time the egocentric viewpoint of the participant's position in the virtual soccer stadium (tracker response latency <10 ms). One metre displacement in the virtual environment corresponded to 1 m displacement in the real environment. The control box for the HMD was mounted on a back pack with adjustable straps. Two 8 m DVI cables connected the HMD control unit to the computer. The position and orientation of the hands in the virtual environment were updated to match those of the participant's hands using signals from two 6 DOF Microtrax Intersense hand trackers (resolution 0.75 mm; 0.05°) attached to a pair of goalkeeping gloves (see Movie S3).

### Procedure

After the participant put on the backpack, the HMD (with head tracker) and the goalkeeping gloves, the virtual environment programme was started and the participant saw himself in the position of a goalkeeper, in the goal-mouth of a virtual soccer stadium. To heighten the level of presence felt in this simulated environment and to provide a sort of visual calibration (see Movie S1), participants were encouraged to walk around for some time and reach out and ‘touch’ a virtual ball suspended in mid-air. They were also encouraged to look at their virtual hands and move them around. Following this period of adaptation, participants were given 20 randomly selected practice trials (not analysed) to become familiar with the task, particularly with respect to the movement amplitudes required to intercept the ball. Saves (or successful interceptions) were defined using a collision detection algorithm programmed in the 3DVia Virtools software (i.e., collision between either of the hands and the ball). Participants were given immediate feedback with the message ‘Great Save’ being displayed on the screen if the ball was saved (see Movie S2). Although before each trial the participant's position in the virtual world was automatically reset to the centre of the goal, for safety reasons a second experimenter was present to reposition the participant in the real space. The actual experiment involved three blocks of 50 trials separated by a 5 minute break. The HMD was always removed during the break and water provided. Ball, head and hand coordinates were recorded at 80 Hz.

### Analyses

In total 16 trials (out of 1800) were omitted from the analyses because one of the hand trackers lost signal and did not transmit data. Hand position was defined as the centre of the virtual hand. Hand movements were filtered using a digital recursive 4^th^-order Butterworth filter. The final error was defined as the lateral position of the hand relative to the ball at the moment the ball entered the goal (whichever hand was closest was used). To calculate the early bias, we first defined the moment of initiation of the movement in the direction of the ball's passing position; the used (absolute) velocity criterion was the maximum of 0.05 m s^−1^ and 2% of the first velocity peak in the direction of the passing position exceeding 0.25 m s^−1^. The early bias was the maximal signed deviation from the initial hand position until the mentioned moment of initiation. To account for differences in initial and final movement planning, the early bias was calculated for the hand other than that used for the final error. The moment of initiation of the entire movement (T_ini_) was defined as the moment relative to the onset of ball motion at which the absolute hand velocity exceeded the maximum of 0.05 m s^−1^ and 2% of the first velocity peak exceeding 0.25 m s^−1^. Note that this T_ini_ is not equal to the one used to quantify the early movement bias, which only referred to the movement component in the direction of the ball's passing position; T_ini_ refers to the entire movement, even if the initial movement was in the wrong direction.

The effects of spin direction and passing position in the novices were analyzed for both variables using a linear mixed model with participant as a random factor (alpha = 0.05). Paired-samples two-tailed *t*-tests were used for post-hoc analyses. The single-subject analyses for the involved univariate ANOVAs, with Student's *t*-tests as post-hoc tests. It should be realized that the power of this single-subject analysis is somewhat lower than the analyses for the novices, resulting in less significant differences in the post-hoc tests. All post-hoc comparisons involved step-down Holm-Sidak adjustment of the original alpha level (alpha = 0.05) for multiple comparisons.

### Aerodynamics model

We used a Newtonian model of aerodynamics, in which three forces operated on the ball: gravity (**F** *_G_*), a drag force (**F** *_D_*), and the spin-induced lift force (**F** *_L_*, also called Magnus-Robbins force).At any time, ball acceleration was calculated from 
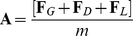
. **F** *_G_* was modelled as 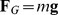, with *m* being 0.43 kg and **g** being 0 is *x*- and *z*-direction, and −9.81 m/s^3^ in the vertical, *y*-direction. **F** *_D_* was defined as:
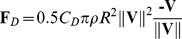
(7)Here, *C_D_* is the drag coefficient (set to 0.33), *ρ* is the air density (set to 1.225 kg/m^3^), and *R* is the ball radius (set to 0.11 m, the official size of a soccer ball). **V** is the instantaneous velocity vector of the ball. The factor 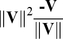 ensures that the **F** *_D_* magnitude scales with the squared ball speed and points in the direction opposite to **V**. Finally, **F** *_L_* was determined according to the Kutta-Joukowski lift theorem [31], [32]. The lift per unit length of a cylinder acting perpendicular to **V** is given by
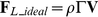
(8)where Γ represents the strength of rotation, defined by 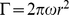. Here, *r* is a variable radius (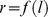). Modelling a sphere as an infinite number of infinitely thin cylinders, the total theoretical ideal lift on a ball (**F** *_L_ideal_*) is defined by the integral
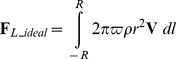
(9)with 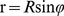 and 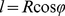 (implying that 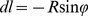), this yields
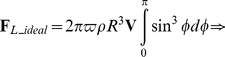


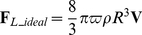
(10)The lift only acts perpendicular to the velocity vector (**V**) and the rotation axis (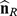), that is, only the component of **V** perpendicular to the 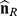 influences the lift (e.g., **F** *_L_ideal_* is maximal for 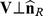 and minimal [zero] for 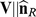). We therefore used the following, more general definition of **F** *_L_ideal_*:
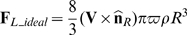
(11)Here, 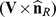 denotes the cross product between **V** and the rotation axis (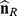). Equation 11 represents the theoretical ideal lift on a spinning ball. However, in reality many additional factors influence the lift, such as configuration of the seams, which are impossible to quantify mathematically. For this reason, the model is extended with a lift coefficient (*C_L_*), which scales **F** *_L_ideal_* to match trajectories observed in reality [32]:
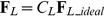
(12)We used *C_L_* = 0.2 in our simulations, resulting in ball trajectories deviating about 2 m from a straight axis for the initial distance of 30 m and ball flight time of 1.4375 s. This *C_L_* was selected to produce a slightly larger curvature than the free-kick shot by Mikael Nilsson in 1993 (see Appendix). This minor exaggeration was deliberate, to maximize the observable effects of spin direction.

### Model Simulations

We simulated ball trajectories by numerically integrating the differential equations of aerodynamics in Matlab (using *ode45.m*). For each trajectory, we optimized the initial ball velocity vector to ensure that the ball passed at the appropriate time, height and lateral position. We fixed the rotation axis during flight to be a vector tilted 15° backwards in a plane defined by the initial velocity vector and gravity. The goalkeeper model was simulated by numerically integrating Equation 6 in Matlab, using ball coordinates derived from the aerodynamics model. The initial hand position was set to −0.15 m for the leftmost passing positions, 0 m for the central passing position, and to 0.15 m for the rightmost passing position (accounting for the minor offset when using the left and right hand). We used *G* = 0.45, *X_heading_max_* = 1.5 m, and *δ_vis_* = *δ_mot_* = 0.05 s. Movements were initiated *δ_vis_* after the experimentally recorded moment of initiation of the novices (Figure 5).

## Supporting Information

Text S1 Analyses for the expert goalkeepers and description of the implications for real goalkeeping.
(0.04 MB DOC)

Movie S1 This movie illustrates the immersive experience the participant gets when interacting with the virtual environment. The head mounted display with attached head tracker ensures that the participant has 360 degree immersion and complete control over where they look, while the hand trackers allow them to have direct control over the position and orientation of the two virtual hands.
(4.28 MB MOV)

Movie S2 This movie is a recording of what the participant sees inside the Head Mounted Display during the experiment. The slight jerkiness is due to the body and head movement as the participant moves to intercept the virtual ball. Note this is not perceptible to the participant as it corresponds to their own ego motion. Likewise, the lower frame rate of the movie (25Hz) affects the image quality, relative to that experienced by the participant inside the HMD (experimental refresh rate 60Hz). For the same reason the simulated spin resulting in the ball's curved trajectory is difficult to make out in this movie (but is very apparent in the HMD).
(4.05 MB MOV)

Movie S3 This movie shows the actual movements made by two participants when intercepting the simulated free-kicks. The first participant is an expert goalkeeper (ExP2), whilst the second participant is a non-expert goalkeeper. The second still at the start of the movie has labels indicating the equipment that is worn by the participant during the experiment.
(5.87 MB MOV)

## References

[1] McBeath MK (1990). The rising fastball: baseball's impossible pitch.. Perception.

[2] Craig CM, Berton E, Rao G, Fernandez L, Bootsma RJ (2006). Judging where a ball will go: the case of curved free-kicks in football.. Naturwissenschaften.

[3] Craig CM, Goulon C, Berton E, Rao G, Fernandez L (2009). Optic variables used to judge future ball arrival position in expert and novice soccer players.. Atten Percept Psychophys.

[4] Bray K, Kerwin DG (2003). Modelling the flight of a soccer ball in a direct free-kick.. J Sports Sci.

[5] Babler TG, Dannemiller JL (1993). Role of image acceleration in judging landing location of free-falling projectiles.. J Exp Psychol Hum Percept Perform.

[6] Brouwer AM, Brenner E, Smeets JBJ (2002). Perception of acceleration with short presentation times: can acceleration be used in interception?. Percept Psychophys.

[7] Gottsdanker RM (1956). The ability of human operators to detect acceleration of target motion.. Psychol Bull.

[8] Rosenbaum DA (1975). Perception and extrapolation of velocity and acceleration.. J Exp Psychol Hum Percept Perform.

[9] Schmerler J (1976). The visual perception of accelerated motion.. Perception.

[10] Maunsell JH, Van Essen DC (1983). Functional properties of neurons in middle temporal visual area of the macaque monkey. I. Selectivity for stimulus direction, speed, and orientation.. J Neurophysiol.

[11] Lisberger SG, Movshon JA (1999). Visual motion analysis for pursuit eye movements in area MT of macaque monkeys.. J Neurosci.

[12] Price NS, Ono S, Mustari MJ, Ibbotson MR (2005). Comparing acceleration and speed tuning in macaque MT: physiology and modeling.. J Neurophysiol.

[13] McIntyre J, Zago M, Berthoz A, Lacquaniti F (2001). Does the brain model Newton's laws?. Nat Neurosci.

[14] Zago M, Bosco G, Maffei V, Iosa M, Ivaneko YP (2004). Internal models of target motion: Expected dynamics overrides measured kinematics in timing manual interceptions.. J Neurophysiol.

[15] Zago M, Bosco G, Maffei V, Iosa M, Ivaneko YP (2005). Fast adaptation of the internal model of gravity for fast interceptions: Evidence for event-dependent learning.. J Neurophysiol.

[16] Dessing JC, Peper CE, Bullock D, Beek PJ (2005). How position, velocity, and temporal information combine in the prospective control of catching: Data and model.. J Cogn Neurosci.

[17] Dessing JC, Oostwoud Wijdenes L, Peper CE, Beek PJ (2009). Adaptations of lateral hand movements to early and late visual occlusion in catching.. Exp Brain Res.

[18] Tarr MJ, Warren WH (2002). Virtual reality in behavioural neuroscience and beyond.. Nat Neurosci.

[19] Savelsbergh GJ, Williams AM, Van der Kamp J, Ward P (2002). Visual search, anticipation and expertise in soccer goalkeepers.. J Sports Sci.

[20] Schlack A, Krekelberg B, Albright TD (2007). Recent history of stimulus speeds affects the speed tuning of neurons in area MT.. J Neurosci.

[21] Bennett SJ, Orban de Xivry JJ, Barnes GR, Lefèvre P (2007). Target acceleration can be extracted and represented within the predictive drive to ocular pursuit.. J Neurophysiol.

[22] Mrotek LA, Soechting JF (2007). Predicting curvilinear target motion through an occlusion.. Exp Brain Res.

[23] Dessing JC, Caljouw SR, Peper CE, Beek PJ (2004). A dynamical neural network for hitting an approaching object.. Biol Cybern.

[24] Dessing JC, Bullock D, Peper CE, Beek PJ (2002). Prospective control of manual interceptive actions: comparative simulations of extant and new model constructs.. Neural Netw.

[25] Montagne G, Laurent M, Durey A, Bootsma R (1999). Movement reversals in ball catching.. Exp Brain Res.

[26] Bullock D, Grossberg S (1988). Neural dynamics of planned arm movements: emergent invariants and speed-accuracy properties during trajectory formation.. Psychol Rev.

[27] Bullock D, Cisek P, Grossberg S (1998). Cortical networks for control of voluntary arm movements under variable force conditions.. Cereb Cortex.

[28] Peper L, Bootsma RJ, Mestre DR, Bakker FC (1994). Catching balls: how to get the hand to the right place at the right time.. J Exp Psychol Hum Percept Perform.

[29] Savelsbergh GJ, Whiting HT, Bootsma RJ (1991). Grasping tau.. J Exp Psychol Hum Percept Perform.

[30] Smith J, Zaal F, Douit S, Fernandez L, Bootsma RJ (2010). Intercepting curving free kicks in football..

[31] (Glenn Research Center website, accessed 2010) http://www.grc.nasa.gov/WWW/K-12/airplane/beach.html

[32] (Glenn Research Center website, accessed 2010) http://www.grc.nasa.gov/WWW/K-12/airplane/balllift.html

