# Supplementary material for: Bending It Like Beckham: How to Visually Fool the Goalkeeper
Source: PLoS One. 2010 Oct 6;5(10):e13161. doi: 10.1371/journal.pone.0013161 (PMC2950839; doi:10.1371/journal.pone.0013161)
Supplement: Text S1 — Analyses for the expert goalkeepers and description of the implications for real goalkeeping. (0.04 MB DOC) [file pone.0013161.s001.doc]

**Bending it like Beckham: How to visually fool the goalkeeper**

Joost C. Dessing, Cathy M. Craig

*Analyses for the expert goalkeepers*

We analyzed the final error and early bias separately for both experts (Figure 4). Like the novices, ball spin induced erroneous initial movements (ExP1: *F*_2,133_ = 81.88; *P* < 10^‑15^; ExP2: *F*_2,135_ = 9.92 < 10^-4^) and final errors (ExP1: *F*_2,133_ = 76.89; *P* < 10^‑15^; ExP2: *F*_2,135_ = 11.24 < 10^-7^) in the direction opposite to the spin‑induced lateral ball acceleration (ExP1: all *P* < 0.001; ExP2, early bias: all *P* < 0.05; final error: difference between CS and CCS significant). These results suggest that the erroneous initial movements for curved ball trajectories occur at all levels of experience.

The early bias of ExP1 varied across passing positions (*F*_4,133_ = 7.85; *P* < 10^‑4^), but it is most informative to consider this effect through the interaction with spin direction (*F*_8,133_ = 19.51; *P* < 10^‑15^). For the central passing position ExP1’s early bias differed significantly between all spin directions, while for CCS free‑kicks it was more leftward than for NS and CS free-kicks for balls passing at 1.5 m and more leftward for CCS than for NS free-kicks for balls passing at 0.75 m (all *P* < 0.001). ExP1 did not show any effect of passing position for NS free‑kicks, while for CS free-kicks the early bias was more rightward when these passed at 0 m, than for those passing at 0.75 m and 1.5 m (*P* < 0.001). For CCS free‑kicks it was more leftward passing at 0 m, 0.75 m and 1.5 m than for those passing at ‑1.5 m, and for balls passing at 0 m and 1.5 m than for those passing at ‑0.75 m (*P* < 0.001). While ExP2 showed substantially smaller early biases that were only influenced by the main effect of spin direction, the early biases of all our participants showed a similar pattern (Figure 4).

The final error of both experts was also affected by the ball’s passing position (ExP1: *F*_4,133_ = 36.39; *P* < 10^‑15^; ExP2: *F*_4,135_ = 11.24; *P* < 10^-5^), showing a general undershoot (both: final error significantly more rightward for balls passing at -1.5 m compared to 0 m, 0.75 m, and 1.5 m, and significantly more leftward for balls passing at 1.5 m compared to 0 m and ‑0.75 m; ExP2: significantly more rightward final error for balls passing at -1.5 m compared to those passing at -0.75 m; all *P* < 0.01). For ExP1 this effect also interacted with the effect of spin direction (*F*_8,133_ = 4.20; *P* < 0.001). For CS free-kicks towards -1.5 m and -0.75 m, final errors were more rightward than for CCS and NS free-kicks (all *P* < 0.001). This effect was mirrored for CCS free‑kicks towards 1.5 m (both *P* < 0.001). The final error differed between CS and CCS free‑kicks towards 0 m (*P* < 0.001). NS free-kicks passing at -1.5 m yielded a more rightward final error compared to the other passing positions (*P* < 0.001), while the final error was also more rightward for CS free-kicks passing at -1.5 m compared to those passing at 0 m, 0.75 m, and 1.5 m, and for those passing at -0.75 m than at 0.75 m and 1.5 m (all *P* < 0.005). Finally, for CCS free-kicks towards 1.5 m the final error was more leftward than for those toward ‑1.5 m, ‑0.75 m, and 0 m (all *P* < 0.01). With the exception of some minor differences, this pattern matches very well with the pattern observed for the novices.

Figure 5 illustrates that the moment of initiation for experts varied similarly as for novices. Both experts showed a significant effect of spin direction (ExP1: *F*_2,133_ = 66.30; *P* < 10^-15^; ExP2: *F*_2,135_ = 10.56; *P* < 10^-4^), while ExP1 also showed an effect of passing distance (ExP1: *F*_4,133_ = 5.07; *P* < 0.001). These effects are discussed here in terms of the spin direction × passing distance interactions (ExP1: *F*_8,133_ = 8.52; *P* < 10^-8^; ExP2: *F*_8,135_ = 11.24; *P* < 10^-11^). ExP1 showed a pattern nearly identical to the novices. For all passing positions, he initiated later for NS than for CS free-kicks (all *P* < 0.005), and earlier for balls passing at ‑1.5 m, -0.75 m, and 0 m for CCS than for NS free‑kicks (all *P* < 0.005). Moreover, ExP1 waited significantly longer for NS free‑kicks towards goal centre (also: later initiation for NS free‑kicks passing at -0.75 m than at 0.75 m; all *P* < 0.005).

ExP2, on the other hand, waited substantially longer for CS free-kicks passing at 1.5 m compared to those passing at ‑1.5 m (*P* < 0.001). For CCS free-kicks he initiated later the further the ball passed to the right (significant differences between passing positions ‑1.5 m and 0.75 m and 1.5 m, and between ‑0.75 m and 0.75 m, and 1.5 m; *P* < 0.001). His movement for CCS free-kicks were initiated significantly earlier than for NS free‑kicks for balls passing at -1.5 m and -0.75 m and for CS free‑kicks compared to NS free-kicks passing at 0 m. Also, movements were initiated significantly earlier for CS free‑kicks passing at 1.5 m compared to NS and CCS free‑kicks. ExP2 thus delayed his movement initiation in those conditions where the novices and ExP1 showed the substantial erroneous early movement biases. His smaller biases in these conditions thus suggest that ExP2 has learned to wait longer for these trajectories, viewing a larger part of the curved ball trajectory before moving more accurately to stop the free‑kick.

*Implications for real goalkeeping*

How well does performance in our task compare to real goal keeping? Part of this question relates to the limited field-of-view of head‑mounted‑displays, as discussed in the main text. The most important observation arguing against this significantly playing into the observed effects of spin direction is the fact that spin-related erroneous movements can actually be observed in real free‑kick situations (see below). Moreover, similar effects were reported for a much wider field‑of‑view; this also refers to the interaction with passing distance [30]. This would suggest that field‑of‑view is not a critical factor. Given that our explanation for the interaction was hypothetical, any effects of field‑of‑view may be evaluated in future tests of this hypothesis.

An additional factor may be that we did not present a kicker in our virtual environment. This was done deliberately to prevent the goalkeeper from inferring the curve direction before the free‑kick is taken [19]. In fact, in real match scenarios the kicker is often occluded by the wall, which means that - as in our study - the goalkeeper is mainly dependent on vision of the ball trajectory (i.e., occluded kicker movements are not very useful). The wall also often blocks the initial part of the ball’s flight path, implying that the needed vision is available even later on in the trajectory than in our experiment. Viewing the ball later leaves less time for movement corrections, and as a result any initial erroneous movement may be more detrimental in the presence of a wall.

We do not claim that these effects occur in all situations with curved free‑kicks. One of our experts indeed showed substantially smaller early movement biases that were almost entirely corrected at interception. In addition, real goal keeping depends on more than just visual information pertaining to the ball (e.g. expectations, for instance based on prior knowledge of the player’s preferred type(s) of free-kick), which potentially can be used to reduce the movement biases. However, our study can explain why even experts sometimes show the erroneous initial movements (which affect performance), particularly when the goalkeeper predominantly depends on visual information about the ball flight.

A clear example of the aforementioned effects was observed in the Champions League match between IFK Göteborg and PSV Eindhoven on March 17^th^ 1993. The player Mikael Nilsson’s extremely curved free-kick prompted the goalkeeper Hans van Breukelen to make a movement error of about 0.8 m to the left before correcting and diving to the right, ultimately missing the ball that would have been within his reach from his initial position [1]. Such erroneous initial movements have also been observed in other matches [e.g., 2-7].

Our most experienced goalkeeper showed much smaller movement biases, which suggests that he was better able to take lateral ball acceleration into account. In real soccer, this may partly come from expectations about the spin direction, inferred from who is taking the free kick and with which foot. However, this strategy could backfire if the goalkeeper puts too much weight on this expectation. This was illustrated by the recent free‑kick of Keisuke Honda at the 2010 FIFA World Cup between Japan and Denmark. Denmark’s goalkeeper, Thomas Sørenson, appeared to expect a clockwise spinning ball that would enter the goal more to the left than its initial heading position. He thus initially moved leftward, only to find out later – too late in this case – that the ball actually was not spinning and thus entered the goal on his right [8]. Earlier in his career, Honda fooled a goalkeeper in a similar manner, leading to an even more extreme erroneous initial movement of the goalkeeper [9]. Another example occurred during the FIFA 2010 World Cup in the Quarter final between Ghana and Uruguay (Diego Forlan [10]).

Because our model pertains to movement control, it does not explain why goalkeepers in certain situations do not move at all (even though these effects are caused by ball spin). For free‑kicks that initially appear to pass wide of the goal posts goalkeepers typically start moving late, and sometimes even do not move at all. A well‑known example of this was observed in a match between France and Brazil on June 3^rd^ 1997 at the Tournoi de France, in which France’s goalkeeper Fabian Barthez hardly moved to the extremely curved free-kick shot by Roberto Carlos, which initially appeared to go wide [11].

**References**

1. (Youtube website, accessed 2010) <http://www.youtube.com/watch?v=ZgNNmDuob-A>
2. (Youtube website, accessed 2010) <http://www.youtube.com/watch?v=adYgHunQEl0&feature=fvst> at 2:20 and 3:30.
3. (Youtube website, accessed 2010) <http://www.youtube.com/watch?v=NgVnwlDQo44> at 5:31.
4. (Youtube website, accessed 2010) <http://www.youtube.com/watch?v=-6xOFL169LQ&feature=related> at 2:22.
5. (Youtube website, accessed 2010) <http://www.youtube.com/watch?v=y3I99_ulZT8&feature=related> at 7:00.
6. (FIFA website, accessed 2010) <http://www.fifa.com/worldcup/highlights/video/video=1266453/index.html> (1-0).
7. (FIFA website, accessed 2010) <http://www.fifa.com/worldcup/highlights/video/video=1269571/index.html> (1-1).
8. (FIFA website, accessed 2010) <http://www.fifa.com/worldcup/highlights/video/video=1257797/index.html> (1-0).
9. (Youtube website, accessed 2010) <http://www.youtube.com/watch?v=0lOZ-n-afY0> at 0:18.
10. (FIFA website, accessed 2010) <http://www.fifa.com/worldcup/highlights/video/video=1266453/index.html> (1-1).
11. (Youtube website, accessed 2010) <http://www.youtube.com/watch?v=Pb2qykj6_ZU>
